# Case Report and Literature Review: COVID-19 and status epilepticus in Dyke-Davidoff-Masson syndrome

**DOI:** 10.12688/f1000research.27971.1

**Published:** 2021-01-08

**Authors:** Lourdes de Fátima Ibañez Valdés, Jerry Geroge, Sibi Joseph, Mohamed Alshmandi, Wendy Makaleni, Humberto Foyaca Sibat

**Affiliations:** 1 Neurology Department, Walter Sisulu University/ Nelson Mandela Academic Hospital, Mthatha, Eastern Cape, 5100, South Africa; 2 Internal Medicine Department, Walter Sisulu University/Nelson Mandela Academic Hospital, Mthatha, Eastern Cape, 5100, South Africa

**Keywords:** Covid-19, Dyke-Davidoff-Mason Syndrome, status epilepticus, hyperglycaemic hyperosmolar syndrome

## Abstract

Dyke-Davidoff-Masson syndrome (DMMS) is a non-inherited rare condition with a clinical constellation of hemiparesis/hemiplegia, facial asymmetry, intellectual disability, and epilepsy. The radiological features can be including unilateral cerebral atrophy, calvarial thickening, and hyper pneumatization of the paranasal sinuses. The condition can either be congenital or acquired. The presentation usually occurs during childhood or early adolescents, but there have been adult cases reported.

Here we report a 48-year-old male who was a known poorly controlled epileptic that contracted SARS-CoV-2 with subsequently developed status epilepticus and, when worked up, was shown to have features of DDMS. This case is unique as the patient had hemiatrophy and epilepsy but managed to lead a normal, physically demanding, and high functioning academic career and presented late in life. Perhaps only due to coronavirus disease 2019 (COVID-19) was this diagnosis picked up.

This report contains a case presenting atypical DDMS in status epilepticus and COVID -19 plus other complications. From our knowledge, this is the first case presenting these comorbidities reported to the medical literature.

## Introduction

In 1580, the first respiratory pandemic was reported ^1^. Up to date, millions of people died, most of them during the 20 ^th^ and 21 ^st^ centuries. The most devastating epidemic and outbreaks were the Spanish Flu (500 million infected) during the early 20th century, even bigger than Hong Kong flu, swine flu, SARS-CoV-1 (2003), and the MERS-CoV outbreak (2012) ^1– 3^. However, the first pandemic causing encephalitis was reported soon after 1580 ^3^.

Since 1965, when human coronaviruses were discovered ^4^, several types of coronavirus (CoV) have been reported, including SARS-CoV-2, SARS-CoV-1, and MERS-CoV, which are all responsible for three epidemics, plus others four types that also infect many human beings (HCoV-229E, HCoV-OC43, HCoV-NL63, and HCoV-HKU1) ^5^. Based on several mechanisms, coronaviruses affect the peripheral and the central nervous system. Even before SARS-CoV-2, other types of coronavirus, such as SARS-CoV-1, HCoV-229E, and HCoV-OC43, also damage the nervous system ^5^.

Wuhan is a large city and the capital of Hubei Province in the People's Republic of China. Wuhan has a population of around 11 million persons. At the beginning of December 2019, an outbreak of many persons presenting viral pneumonia of an unknown agent was reported. In the following month (January 7, 2020), some Chinese authors identified the etiological agent of that respiratory disease and called it by 2019-nCoV (for 2019 novel coronavirus) ^6– 8^.

Since December 2019, documented an increasing number of cases presenting the novel coronavirus disease of 2019 (nCOVID-19) and associated neurological manifestation are published every month. CoV also caused neurological lesions like anosmia and ageusia with different prevalence in China (5%) ^6^ or in Italy (88%) ^9^.

In a recent study by Dorche *et al*., the following list of neurological complications were observed: headache and dizziness (the most common on initial presentation), fatal encephalitis with HCoV-OC43 (two immunosuppressed infants), acute disseminated encephalomyelitis (one 15-year-old boy with HCoV-OC43 and four adults with SARS-CoV-2), acute flaccid paralysis (HCoV 229E) and OC43 (one 3-year-old girl), ischemic (1.3%) and hemorrhage (0.5%) strokes, encephalitis with SARS-CoV-1 RNA (one 39-year-old patient), different presentation of Guillain-Barré syndrome, cerebral venous sinus thrombosis (13 patients in nine studies), acute encephalomyelitis (four patients), acute myelitis (five patients), optic neuritis (one patient) altered level of consciousness (nonconvulsive status epilepticus, infections, parenchymal lesions, electrolyte disturbances, hypoxic, toxic and metabolic encephalopathies), leukoencephalopathy (18 patients in three studies), acute necrotizing encephalopathy (eight patients), other encephalitis (22 patients out of 13 reviews), mild encephalitis/encephalopathy with a reversible splenial lesion(MERS), posterior reversible encephalopathy syndrome (PRES), and Bickerstaff's encephalitis (BBE), ^10– 14^. In patients infected by SARS-CoV-1 and SARS-CoV-2, epileptic seizures have been reported ^13^ also in infected patients with MERS-CoV ^12^ and SARS-CoV-2. In COVID-19, (48 epileptic patients out of 20 studies), visual impairments (12 patients out of 3 reviews), impaired eye movement mainly due to Abducens nerve palsy (12 patients out of 4 reviews), trigeminal neuropathy (in 9 patients out of 2 studies), Miller-Fisher syndrome (52 patients out of 36 studies), skeletal muscle injury and muscular diseases Have been reported ^10^.

Nepal and colleagues ^15^ reported one case of Bell's palsy, as a neurological presentation of COVID-19. Another group of authors published two new patient cases but did not include enough supporting information to draw firm conclusions ^16^. At the same time, other authors have published cases presenting generalized epileptic seizures ^17– 22^.

One case of focal status epilepticus (SE) was reported by Vollono *et al*. ^23^ and acute epileptic encephalopathy by others ^24– 26^, including the treatment for these conditions ^27^.

A systematic review done by Ghannam *et al*. found two cases of SE, one of which had a past medical history of epilepsy from another cause ^28^. Gelisse *et al*. established that some patients with severe SARS-CoV-2 infection are at risk of subclinical epileptic seizures or even nonconvulsive status epilepticus (NCSE) and recommend video EEG monitoring in some cases ^29^.

Recently, some authors have speculated that acute epileptic seizures may be due to swelling of the brain cortex (encephalitis) and the direct damage of the brain cortex by the virus because SARS-CoV-2 can be present in the cerebrospinal fluid (CSF) of some patients ^18, 30, 31^.

In other extensive studies involving several hundreds of COVID-19 patients, the authors concluded that none of their cases had acute symptomatic seizures or SE ^20, 32– 42^.

Nevertheless, the retrospective case series published by Somani and collaborators ^25^ deserves special mention. These investigators published the electroencephalographic findings and clinical manifestations of two COVID-19 patients with new-onset SE without a previous history of epilepsy or acute epileptic seizures. Both patients had SARS-CoV-2 pneumonia confirmed by CT scan and PCR; however, the authors did not perform CSF and could not rule out meningoencephalitis. The second patient presented a new-onset refractory status epilepticus. The same author established the neurovirulence of SARS-CoV-1, finding the presence of viral antigen in the thalami, hippocampus, medulla oblongata, and mesencephalic regions that regulate cardiorespiratory functions in a human autopsy series ^25^. Some recent good news is the excellent response of SE to levetiracetam reported by two investigators ^24, 25^.

Other investigators also recommend the use of verapamil in patients presenting SE stage III and SARS-Cov-2 infection ^43^. The same authors reported the first patient affected by PRES and SARS-CoV-2 without SE. In contrast, Mohammad *et al*. wrote about a 32-year-old male with tonic-clonic generalized SE ^44^. In the meantime, other investigators delivered essential recommendations to improve the management of SE during the pandemic despite the lack of ventilators and ICU facilities ^45^.

Acquired or congenital (infantile) cerebral hemiatrophy, otherwise referred to as Dyke-Davidoff-Masson syndrome (DDMS), was first described in 1933 by Dyke and colleagues ^46– 48^. DDMS is a non-inherited rare condition ^49^, with an unknown frequency; most of the literature stems from either case reports or series ^50^. DDMS is a diagnostic constellation made up of hemiparesis/hemiplegia, facial asymmetry, intellectual disability, and treatment-resistant epilepsy, classically with distinct neuroimaging features ^48^. However, according to Ayaz *et al*., the syndrome has varied clinical and radiological spectrum presenting at different life stages ^51^. The classical imaging findings are hypoplasia of one brain hemisphere (hemiatrophy), often accompanied by volume reduction of corresponding cranial fossa and thickening of nearby bony structures and equilateral enlargement paranasal sinuses, the frontal sinus being the most involved or hyperpneumotisation of mastoid air cells. The congenital type can be due to insults suffered during fetal or early childhood development, such as ischemia, trauma, infarction, hemorrhage, and infections. However, the acquired type is usually associated with trauma, infectious diseases, or hemorrhages after one month of age ^47^. We know that hemispherectomy is the best treatment for patients who have drug-resistant and disabling seizures.

At the time of writing, the coronavirus disease-19 (COVID-19) pandemic continues infecting peoples worldwide. COVID-19, caused by SARS-CoV-2, has thus far claimed 23,057,288 cases worldwide ^52^ and 607 045 patients in South Africa ^53^. Up to date, 46 medical doctors died in the Eastern Cape province alone.

We performed an extensive search of the medical literature to answer our research question: "What is the reported frequency of status epilepticus in patients with DDMS and coronavirus infections?

## Case presentation

A 48-year-old African male patient was admitted to Nelson Mandela Academic Central Hospital (NMACH) in Mthatha, South Africa. He was born out of a non-consanguineous marriage and was referred from a regional hospital with tonic-clonic-generalized status epilepticus. On initial presentation to the base hospital, he was given diazepam 10 mg IV stat (dose repeated twice) and then loaded with phenytoin 750 mg.

This patient had a past medical history of chronic epilepsy for many years but was well-controlled on valproate acid CR 500 mg PO Bd, levetiracetam 750 mg PO BD per day. There was no facial asymmetry, no hemiplegia, the rest of the cognitive functions were average, and there are no mental retardation signs.

He was also a chronic hypertensive. He worked as a police officer on further inquiry and he did not smoke, consume drugs or alcohol. We did not obtain remarkable information on birth history, developmental milestones, education history, prior admissions to hospital, and childhood illnesses.

We found no noticeable body asymmetry on examination. The patient had pink mucous membranes, was well hydrated, and afebrile with a GCS 11/15 (E3V3M5); his motor examination revealed a power 3/5 with spastic hypertonia on left upper and lower limbs, and no fits noted.

He was in respiratory distress with tachypnea of 30 breaths/minute saturating at 84% on a 40% venture face mask. The rest of the vital signs showed a BP 118/88 mmHg and Pulse 98 bpm. He had scattered crepitation on the chest bilaterally. Table 1 shows all blood test results.

**Table 1.** Blood test results.

Blood test variables	Patient value	Normal range
White cell count	5.30 × 10 ^9^/L	3.9–12.6 × 10 ^9^/L
Hb	16.1 g/dL	12–15 g/dl
Platelets	365 × 10 ^9^/L	186–454×10 ^9^/L
Sodium	141 mmol/L	136 – 145 mmol/L
Potassium	3.8 mmol/L	3.5–5.1 mmol/L
Chloride	113 mmol/L	98–105 mmol/L
Urea	8.8 mmol/L	2.1–7.1 mmol/L
Creatinine	122 µmol/L	48–90 µmol/L
Calcium	2.20 mmol/L	2.15–2.5 mmol/L
Magnesium	0.87 mmol/L	0.63–1.05 mmol/L
Phosphate	1.40mmol/L	0.78–1.42 mmol/L
C–reactive protein	23 mg/L	<10 mg/L
Erythrocyte sedimentation rate	12 mm/hr	0–10 mm/hr
Total protein	72 g/L	60–78 g/L
Total Bilirubin	<4 µmol/L	5–21 µmol/L
Alkaline phosphatase	90 U/L	42–98 U/L
Aspartate transaminase	23 U/L	13–35 U/L
Alanine transaminase	19 U/L	7–35 U/L
Total cholesterol	4.78 mmol/L	<4.5 mmol/L
HbA1C	5.1%	<7%
Valproate level	427 µmol/L,	346.70–693.40 µmol/L
Phenytoin level	139 µmol/L	20–40 µmol/L

Serum levels of interleukine-6 were not available. PCR confirmed SARS-CoV infection, but we did not perform ferritin and procalcitonin investigations.

**Figure 1.**
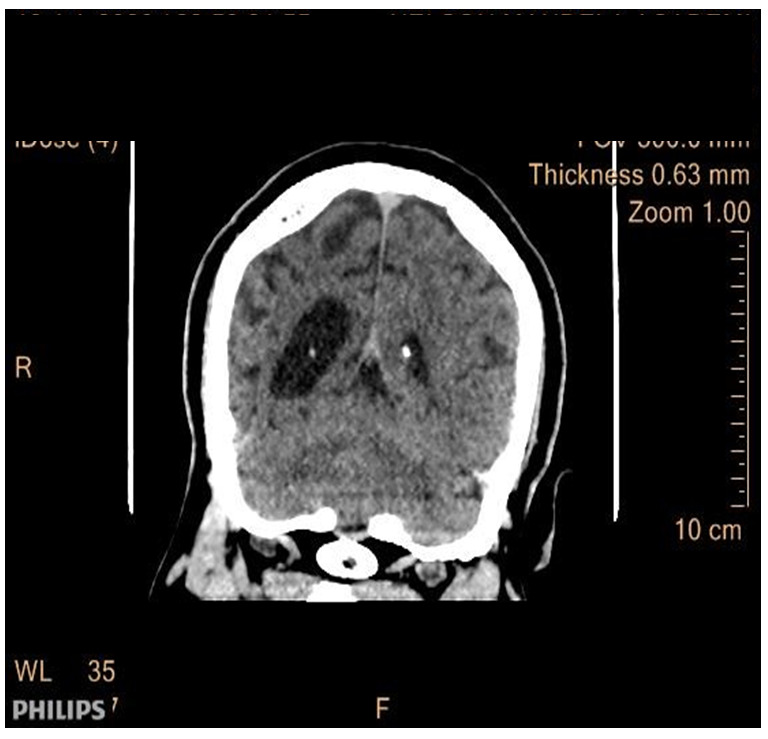
CT scan of the brain (coronal view). Shows a notable atrophy of the right cerebral hemisphere with enlargement of the ipsilateral lateral ventricle.

**Figure 2.**
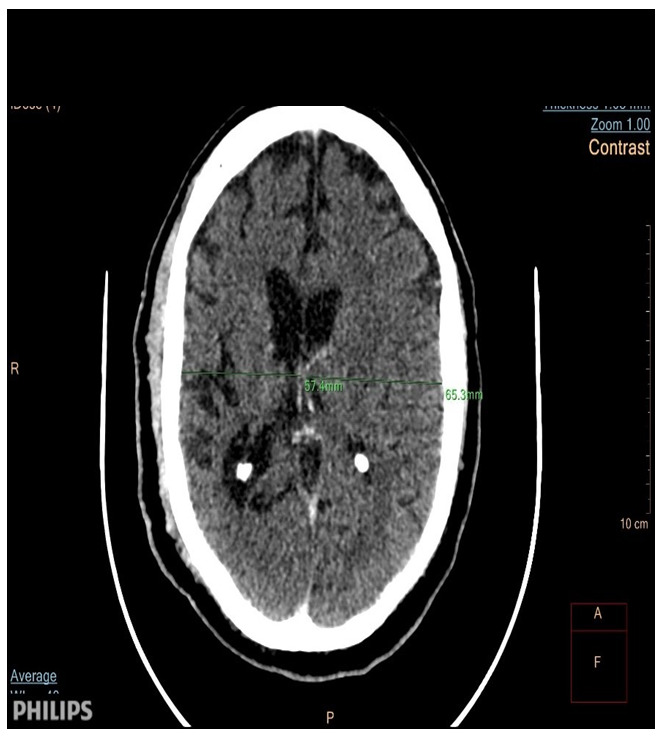
CT scan of the brain (axial view). Shows asymmetry of the lateral ventricles (right to left) with a notable atrophy of the right cerebral hemisphere.

**Figure 3.**
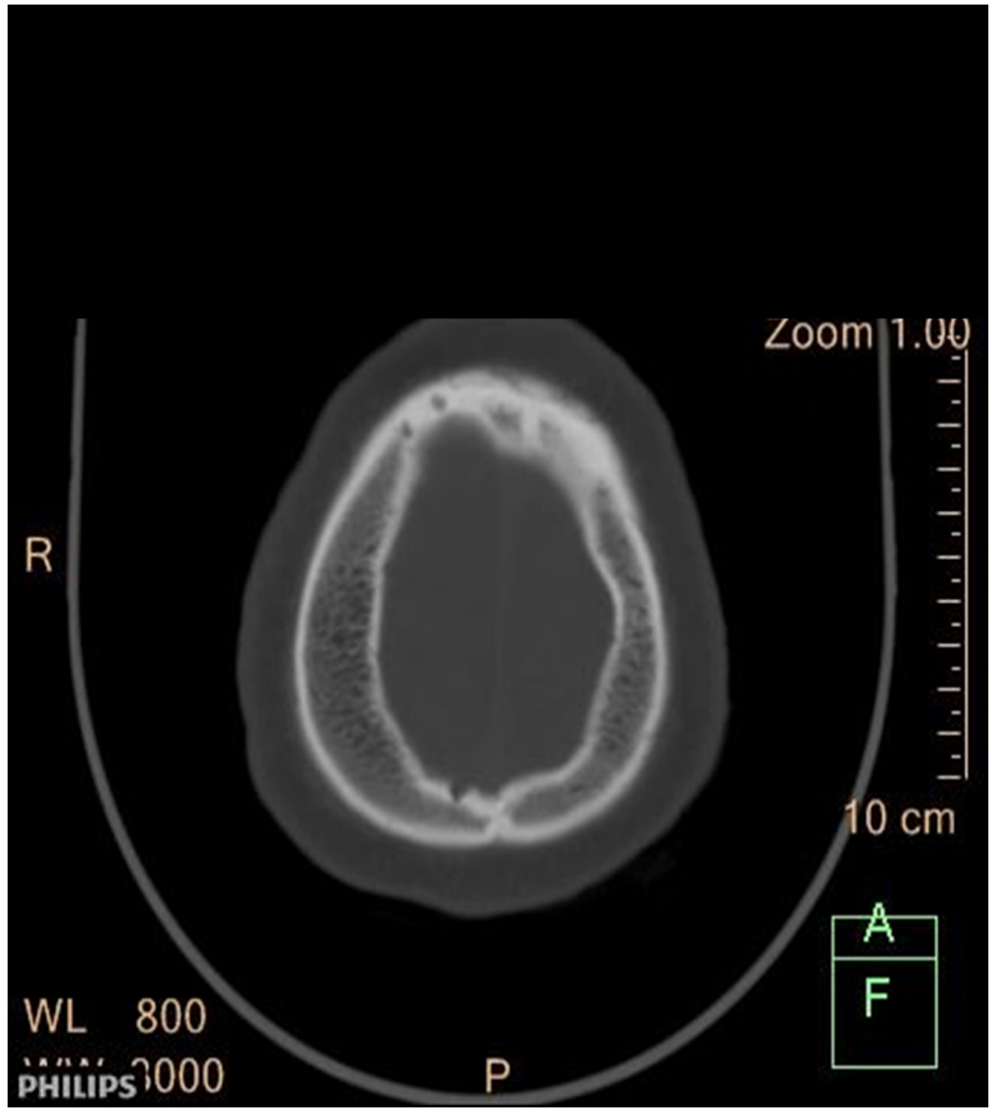
CT scan of the head (axial view). Shows a marked thickness on the right side of the skull.

The patient presented with a one-day SE type II (established), characterized by recurrent tonic-clonic generalized seizures with impaired awareness. As he did not recover, progressive doses of anti-seizure medication (ASM) were administered, reaching a total of 20 mg of diazepam (10 mg IV twice), 1500 mg of phenytoin (IV bolus), and 1500 mg of valproate (25 mg/kg), without recovering. An urgent cranial CT scan of the brain revealed atrophy on the right cerebral hemisphere with associated thickening of the calvarium on the same side without hyperpneumotisation of paranasal sinuses or mastoid air cells ( Figure 1, Figure 2 and Figure 3), suggestive of Dyke-Davidoff-Masson Syndrome; otherwise, there was no bleed or area of infarct, and there was no space-occupying lesion.

On the second day of admission, the patient was admitted to the COVID ward, put on high-flow nasal oxygen (60% at 15 L/min), dexamethasone 8 mg IV daily, Clexane 60 mg SC 12-hourly, ceftriaxone 1 g IV every day, azithromycin 500 mg Po daily, vitamin D 50000 U PO weekly, vitamin C 250 mg PO 8-hourly, diazepam 10 mg IV if fitting, valproate 500 mg IV 12-hourly, phenytoin 100 mg IV 8-hourly, amlodipine 10 mg orally daily, Ridaq 25 mg orally daily and intravenous fluids (1 L Ringers lactate IV 8-hourly).

After two days of admission, the patient improved neurologically and presented no more seizures, but his respiratory distress continued progressively getting worse, and his septic markers were rising. Two days later, blood levels showed values of Na 159 mmol/L, K 5.1 mmol/L, urea 42 mmol/L, creatinine 275 µmol/L, CRP 117 mg/dL, T protein 88 g/L, Alb 38 g/L, ALT 48 U/L, AST 168 U/L, GGT 190 U/L, ALP 58U/L, white cell count 14.30 × 10 ^9^/L, Hb 17.1g/dl, platelet count 316 × 10 ^9^/L cholesterol level no done, Blood gas showed Ph. 7.46, PaCO _2_ 36 mmHG, PaO _2_ 66mmHg, HCO _3_ 27mmol/L, Na 160 mmol/L, K 3.5 mmol/L, Ca 1.05 mmol/L, Hgt 25.6 mmol/L, blood oxygen saturation 84%. The patient's urine did not contain ketone bodies.

On the fifth day after admission, the patient was assessed as having ARDS secondary to COVID-19 and hyperglycemic hyperosmolar state (HHS), and high-flow nasal O _2_ was increased to 100% concentration at 20 L/min. One and a half hours after the onset of the symptoms, the patient had not recovered yet. The patient began to fit again and under the suspicion of refractory status epilepticus secondary to HHS and neuro-COVID 19; when another round of 20 mg of diazepam (10 mg IV twice), 1500 mg of phenytoin (IV bolus), and 1500 mg of valproate (25 mg/kg) was started, the patient developed cardiac arrest and demised.

## Discussion and literature review

Our literature review utilized the Preferred Reporting Items for Systemic review and Meta-Analysis statement. However, we did not conduct a classical systematic review.

We reviewed the databases published before August 20, 2020, such as Medline EMBASE, Scopus online databases, Google Scholar, to identify articles evaluating COVID-19 and SE in DDMS. All items about "neurologic complications* OR epilepsy* OR brain* OR status epilepticus* OR fits* OR neuronal lesion* OR Neuro-Covid* OR cortical lesions* OR DDMS OR * OR seizure* OR COVID-19* OR unconsciousness* OR acute epileptic seizure*, OR Duke Davidoff Mason Syndrome*" where * is the PubMed wildcard for every possible word beginning or ending. Other neurological combinations were considered beyond the scope of the current work and no included. Finally, we did not find a publication related to COVID-19, SE and DDMS.

Our patient complained of chronic arterial hypertension, and this condition and diabetes mellitus is associated with a significant risk of lung disease leading to COVID-19 severity. Despite the patient's condition, antihypertensive therapy should continue in COVID-19 patients ^54^. Concerning our patient, it's important to highlight that diabetes mellitus by itself is one of the most relevant comorbidities associated with the severity of all coronavirus infections, including the current SARS-CoV-2, and affected cases have an increased risk to develop severe complications such as acute respiratory distress syndrome and systemic organ failure ^55^. COVID-19 patients with hyperglycemia are at risk of developing other infections, including influenza and pneumonia with increasing mortality rate; this is also applicable to other SARS coronavirus, pandemic influenza A 2009 (H1N1), and middle east respiratory syndrome coronavirus ^56– 59^.

Here we discuss elevated urea and creatinine in our patient. Therefore, it is essential to mention that apart from diabetes and hypertension, acute kidney injury has also been documented in some patients with COVID-19. *ACE2* gene expression in renal cells and bladders cells has been investigated, and the results confirmed damage of the renal proximal tubule cells and the bladder epithelial cells by COVID-19 infection ^60^. SARS-CoV-2 affects the kidneys ^61^, which has been confirmed by examining viral nucleocapsid protein accumulated in the renal tubules by post-mortem examination proved that ^62^.

DDMS is due to atrophy of one cerebral hemisphere and usually occurs due to an insult to the brain *in utero* or an early period of childhood ^63^. In the first description, Dyke, Davidoff, and Masson described nine patients who had a constellation of seizures, facial asymmetry, mental retardation, and hemiparesis with bare skull X-ray changes (ipsilateral osseous hypertrophy and calvarial thickening) ^64^. We can understand intracranial pathology with MRI and CT scans, which results in such clinical presentation. Our patient is atypical because he did not complain of weakness on the left half body, was strong enough to work as a police officer, and there was no evidence of mental retardation was observed.

Some patients with DDMS can complain of psychiatric manifestations in rare instances ^65^. The radiological features can include unilateral cerebral atrophy, calvarial thickening, and hyperpneumotisation of the paranasal sinuses ^66^. As with our patient, mental retardation does not always need to be present, and the seizures may develop years after the initial insult ^67^.

Some authors classify DDMS as either congenital/primary or acquired/secondary. The congenital form occurs due to an insult that happens *in utero*. It could be infections or vascular disorders occurring during the gestational period (unilateral cerebral artery pathologies or mid aortic arch coarctation) ^68^. The congenital forms usually present during the perinatal period. The acquired form occurs due to early childhood infections, trauma, tumors, asphyxia, intracranial ischemia, or hemorrhage.

To better understand the anatomical changes occurring, it is crucial to understand the brain's growth and surrounding structure. The mail sulci form around 3 months’ gestation up to approximately eight months of pregnancy. If there are no prominent sulci visible on imaging, the congenital form of DDMS is present. Most brain and skull development occurs during the first three years of life (reaching 75% of adult size). The outward pressure of the brain parenchyma on the skull contributes to this growth. If there is unilateral atrophy, then the surrounding structures will grow inwards (calvarial thickening, enlarged sinuses, increased width of diploid spaces ^69, 70^. Hangmen *et al*. proposed that the congenital form of DDMS be named unilateral cerebral hypoplasia because there is hypoplasia instead of atrophy ^71^.

A literature review done by Unal *et al*. showed that in the pediatric presentations, there is a male predisposition towards DDMS, and the left hemisphere is more commonly affected; the mean age at diagnosis was 11 in this review ^72^. However, a literature review done by Diestro *et al*. in 2018 comprising 21 patients with a mean age at presentation being 31 years old showed a slight female despondence, and these adult presentations more commonly involving the right cerebral hemisphere. In 28% of cases (6/21), there was no mental retardation, and in 14% (3/21), it was unknown whether there was mental retardation ^73^.

The association of SARS-CoV and seizures was known even before the current pandemic. In 2003 authors published the first observed case ^74^. The following year, other patients were reported ^75^, and later another case was associated with a different coronavirus ^76^. However, the total number of reported cases is small. SE and encephalopathy have been reported in children as a presentation of COVID-19. The mechanism of production of seizures is also known ^77^. These authors ^77, 78^ proposed that epileptic seizures can be due to several mechanisms, such as direct infection of the virus, a post-infectious mechanism, an autoimmune response, hematogenous pathway and thrombosis ^79^, by dysregulated cytokine storm ^79^, and by the retrograde neural way, hypoxia, and via the ACE-2 enzyme ^80^.

Association between DDMS and SE is hugely uncommon, and before the current COVID-19 pandemic, only three adolescent patients have been reported: one in 2015 ^81^ and the other two in 2018 ^82^. Other recent systematic reviews, case series, and case reports did not mention any association of DDMS and SE ^51, 63, 73, 83– 90^. At the time of writing (August 25, 2020), no patients presenting DDMS and SE infected by COVID-19 have been reported to the medical literature.

Differential diagnoses, such as Silver-Russell syndrome, basal ganglia germinoma, neurofibromatosis, Parry-Romberg Syndrome, Sturge-Weber syndrome, Rasmussen encephalitis, Fishman syndrome, linear nevus syndrome, and Rasmussen encephalitis, should be considered during the management of these patients.

Finally, we want to highlight some precautions to be considered when treating patients in SE and COVID-19. As aforementioned, DDMS causes epilepsy, epileptic seizures, and even SE. On the other hand, COVID-19 can also cause epileptic seizures and SE. However, the use of some ASM and anti-COVID medicines may cause complications. Therefore, some medications should be used with caution. For example, lacosamide is recommended for the adjunctive treatment of partial-onset seizures, diabetic neuropathic pain, and to control attacks in refractory SE. However, it can prolong the PR interval on an electrocardiogram. Hydroxychloroquine extends the QT interval ^91^. Elongation of the QT interval can also be caused by azithromycin, phenytoin, carbamazepine, and rufinamide, leading to cardiac conduction disturbances ^13^. Combining ASM with hydroxychloroquine and azithromycin can be harmful. Therefore, we recommend EKG monitoring.

## Conclusion

In our opinion, the SE in our patient had a multifactorial origin, including HHS and atypical DDMS. This could have created a susceptible environment in which the new coronavirus's disease acted as a SE trigger. These hypotheses make this case a unique report.

To our knowledge, this patient is the first case of SARS-CoV-2 infection leading to TCG-SE type 2 on a DDMS patient published in the medical literature.

## Data availability

All data underlying the results are available as part of the article and no additional source data are required.

## Consent

Written informed consent for publication of their clinical details and clinical images was obtained from the relatives of the patient.
