# Supplementary material for: Case Report and Literature Review: COVID-19 and status epilepticus in Dyke-Davidoff-Masson syndrome
Source: F1000Res. 2021 Jan 8;10:9. [Version 1] doi: 10.12688/f1000research.27971.1 (PMC9214268; doi:10.12688/f1000research.27971.1)
Supplement: Peer review history

# Peer review: Reviewer response for version 1

**DOI:** 10.5256/f1000research.30937.r134971

**Published:** 2022-06-21

**Authors:** Rizwana Shahid

**Affiliations:** 1 Section of Neurology, College of Medicine, Imam Abdulrahman Bin Faisal University, Dammam, Saudi Arabia

It is an interesting case, highlighting a rare disease with the co-occurrence of the current pandemic. As we are aware, any infection can precipitate status epilepticus in epileptic patients, but with the emergence of the new COVID-19 virus, this report gives the reader good insight.

 In this patient, the reason for status epilepticus is multifactorial rather than being secondary to only DDMS/COVID-19 infection, and the authors have addressed this issue in their discussion.

 There is one issue that needs to be addressed:

- The patient was chronic epileptic, as per the authors, and was on many AED but there is no imaging done before. Can you explain why the patient wasn’t worked up before?

Are enough details provided of any physical examination and diagnostic tests, treatment given and outcomes?

Partly

Is the case presented with sufficient detail to be useful for other practitioners?

Yes

Is sufficient discussion included of the importance of the findings and their relevance to future understanding of disease processes, diagnosis or treatment?

Yes

Is the background of the case’s history and progression described in sufficient detail?

Yes

Reviewer Expertise:

NA

I confirm that I have read this submission and believe that I have an appropriate level of expertise to confirm that it is of an acceptable scientific standard.

# Peer review: Reviewer response for version 1

**DOI:** 10.5256/f1000research.30937.r76973

**Published:** 2021-01-29

**Authors:** Emilia Virginia Noormahomed

**Affiliations:** 1 Department of Microbiology, Universidade Eduardo Mondlane (UEM), Maputo, Mozambique

This is an interesting and unique case report presentation documenting a COVID-19 and status epilepticus in Dyke-Davidoff-Masson syndrome, its management and outcomes. It also discusses and highlights some precautions to be considered when treating patients in SE and COVID-19.

 The infection by SARS-COV-2 is a very recent disease which possesses many challenges in terms of diagnosis and clinical management of its less common clinical presentation. This case report and review of the literatures provide scientific information that will enrich the few existing data, thus contributing to better understand the disease and associated co-morbidities, especially in case of associated neurological diseases.

 Further, this case report is of potential importance to a global audience. My suggestions for further improvement are as follows:

1. Make corrections to language and grammar in the manuscript.
2. Spell out some acronyms the first time they write it.

Are enough details provided of any physical examination and diagnostic tests, treatment given and outcomes?

Yes

Is the case presented with sufficient detail to be useful for other practitioners?

Yes

Is sufficient discussion included of the importance of the findings and their relevance to future understanding of disease processes, diagnosis or treatment?

Yes

Is the background of the case’s history and progression described in sufficient detail?

Yes

Reviewer Expertise:

Infectious diseases and health professionals education

I confirm that I have read this submission and believe that I have an appropriate level of expertise to confirm that it is of an acceptable scientific standard.

# Response

**Published:** 2021-01-29

**Authors:** Humberto Foyaca-Sibat

**Affiliations:** Walter Sisulu University, South Africa

Dear Prof Noormahoomed

 Thanks a lot for your review and constructive suggestions. We will correct by what you suggested.

 Again, thanks for your kind attention and professionalism

 Regards

 Dr Foyaca
